# The Impact of Computed Tomography Scans on the Management and Wait Times in Perianal Abscess Diagnoses

**DOI:** 10.7759/cureus.49417

**Published:** 2023-11-26

**Authors:** Raja Gnanadev, Aldin Malkoc, Alexandra Nguyen, Tara Weaver, Olga Lebedevskiy, Farabi Hussain, Edwin Kim

**Affiliations:** 1 General Surgery, Arrowhead Regional Medical Center, Colton, USA

**Keywords:** cost, radiation exposure, general surgery, emergency department, computed tomography, perianal abscess

## Abstract

Background

Diagnosis and management of perianal abscesses (PAA) are based on history and clinical examination. Imaging is not indicated except in complicated cases, as determined by the surgical team. The monetary, ionizing radiation, and resource utilization costs of a computed tomography (CT) scan in the emergency room must be considered when used for diagnostic purposes of PAAs.

Methods

A retrospective analysis of 129 patients diagnosed with a diagnosis of PAA between 2015-2020 was performed. The primary endpoints included length of stay, CT performed, time from patient presentation to CT, and CT scan completion prior to surgical consultation. Data is reported as n (%) or median (IQR).

Results

Of the 129 patients diagnosed with PAA, 81 underwent CT, and 48 did not. General surgery was consulted in 88% of cases. There were no statistically significant differences in age (p=0.562), sex (p=0.531), or ethnicity (p=0.356). The median hospitalization time was two days when CT was performed (p=0.001). The median time elapsed from presentation to the emergency department and CT scan performed was 16 hours (p=0.001). CT scans were ordered before the surgical consultation in 65% of cases (p=0.001) and 17% after a surgical consultation was placed (p=0.009).

Conclusion

Performing CT scans prior to surgical evaluation for the diagnosis of PAA is not a responsible practice. The cost, resources, and radiation exposure must be considered. This study demonstrated that more CT scans are ordered prior to surgical consultation for PAA, resulting in a prolonged wait time in the emergency department.

## Introduction

Perianal abscess represents a subtype of anorectal abscess that is specifically found in the subcutaneous tissue of the anal verge. Previous studies have suggested that anal gland obstruction causes 90% of perianal abscesses, with only 10% attributed to other causes, such as Crohn's disease, trauma, and human immunodeficiency virus [1]. It is estimated that there are approximately 100,000 cases per year in the United States [2]; however, perianal abscesses are likely underreported and incorrectly diagnosed as hemorrhoids [3]. The imprecise incidence rate of perianal abscesses may also be attributed to single-institution publications and variations in definitive treatment: physician offices, emergency rooms, outpatient surgery centers, and operating rooms.

Historically, some have argued that computed tomography (CT) scans are beneficial in the diagnosis of perianal abscesses, as CT scans are readily available, cheaper than the cost of hospital admission, and useful in the diagnosis of complications such as anorectal sepsis [4]. The reported sensitivity of CT for anorectal abscess is 77% compared to endoanal ultrasound and surgical findings [5]. A systematic review of randomized controlled studies performed in 2017 determined that a thorough history and physical examination are sufficient for the diagnosis of perianal abscess [6]. Further CT imaging should be performed when a complex fistula, complex abscess, or recurrent episodes of perianal abscess are suspected. Obtaining CT images prior to surgical consultation may delay diagnosis, as patients' time in the emergency department may be extended due to waiting for imaging orders, completing imaging, and reading by a radiologist. Radiation exposure, cost, and resources should also be considered. We sought to evaluate whether CT scans in patients with a final diagnosis of perianal abscess changed management in those who presented to the emergency department at a single county institution. This article was previously presented as a meeting abstract at the Annual American College of Surgeon Clinical Congress on October 17th, 2022.

## Materials and methods

This study is a retrospective chart review focused on patients from 2015 to 2020 at a county hospital in San Bernardino County, California. An inquiry into the electronic health records of patients who underwent a computed tomography (CT) of the abdomen and pelvis in the emergency department with a discharge diagnosis of perianal abscess was performed. This yielded a total of 129 patients.

Parameters included age, sex, ethnicity, length of hospitalization, the time at which a CT was ordered, the time at which the same ordered CT scan was performed, whether a CT scan was performed before or after a surgical consultation, and various medical comorbidities. The study considered the frequency of abdominal CT scans being performed in patients with a discharge diagnosis of perianal abscess. The final sample size (n=129) included 81 records of individuals who had a CT scan performed prior to surgical evaluation and 48 who first had surgical consultation for the diagnosis of perianal abscess. Basic demographic and clinical information were also retrieved. The exclusion criteria were as follows: pediatric patients (age <18 years), pregnant women, and those who were comatose or cognitively impaired. This study was approved by the Arrowhead Regional Medical Center's Institutional Review Board #20-47. This study was conducted in compliance with the ethical standards of the institution responsible for human subjects as well as with the Helsinki Declaration.

The samples were considered in two cohorts: subjects who underwent a CT scan for a probable diagnosis of perianal abscess and patients who had a surgical consult prior to a CT scan. Univariate statistics for the categorical variables were assessed using the student's t-test and Chi-squared test, where indicated. Continuous non-parametric variables were analyzed using the Mann-Whitney U-test. Data were compiled into an Excel spreadsheet (Microsoft, Redmond, Washington) and statistical analysis was performed using the Statistical Package for the Social Sciences (SPSS) version 27.0 (IBM Inc., Armonk, New York), unless otherwise indicated. Statistical significance was set at p<0.05.

## Results

We sought to determine the clinical characteristics associated with CT use for the diagnosis of perianal abscess. In the study sample (n=129), the median age of patients in the group without CT was 42 years, and the median age of patients with CT was 42 years (p=0.562). Among those without CT, 81% were male. Of those with a CT performed, 77% were male (p=0.531). Ethnicity groups were also considered, with 65% being Hispanic in the group without a CT performed and 48% in the group with a CT performed (p=0.356). The median risk factor for the group where a CT was not performed was 0.5, and 0.0 in the group where a CT was performed (p=0.991); the remainder of the results are highlighted in Table 1.

**Table 1:** Sociodemographic and patient characteristics with and without perianal abscess diagnosis by CT in the emergency setting ^a^analyzed with non-parametric analysis. All other comparisons with the Chi-squared test. Categorical variables are represented as a total number with percentages. Non-parametric data is presented as a medium with an interquartile range.

Characteristic	CT not performed (n=48)	CT performed (n=81)	P-value^a^
Age^a^	42 (33, 51)	42 (37, 53)	0.562
Male (%)	39 (81%)	62 (77%)	0.531
Ethnicity	-	-	0.356
Hispanic (%)	31 (65%)	48 (59%)	-
African-American (%)	11 (23%)	14 (17%)	-
White (%)	6 (13%)	19 (23%)	-
Total medical comorbidities	0.5 (0, 1)	0.0 (0, 1)	0.991
Diabetes mellitus (%)	15 (31%)	26 (32%)	0.920
Hypertension (%)	14 (29%)	24 (30%)	0.956
Hyperlipidemia (%)	7 (15%)	11 (14%)	0.874
Chronic kidney disease (%)	4 (5%)	3 (6%)	0.751
Congestive heart failure (%)	0	1 (1.2%)	0.440

Table 2 presents the patient outcomes when CT was or was not performed. The median length of hospitalization in the group with CT performed was 2.0 days and 0.0 in the group without CT performed (p=0.001). The median time until CT was performed was 16 hours (p=0.001). Of all the patients who were diagnosed with perianal abscess and who underwent CT performed, 67 had a surgical consult placed (p=0.009). In the group where CT was performed, 65% of CT was ordered prior to surgical consultation (p=0.001), and 17% were ordered after a surgical consultation (p=0.009). Sixty-eight percent of patients who had a CT performed compared with 43% who did not undergo a CT performed followed up in the surgery clinic (p=0.007). When considering whether the patient was discharged from the ED versus the hospital, 42% were discharged from the ED if they underwent a CT performed, and 85% were discharged if they did not undergo CT performed (p=0.001).

**Table 2:** Patient characteristics and outcomes with perianal abscess diagnosis ^a^analyzed with non-parametric analysis. All other comparisons with the Chi-squared test. Categorical variables are represented as a total number with percentages. Non-parametric data is presented as a medium with an interquartile range.

Characteristics/outcomes	CT not performed (n=48)	CT performed (n=81)	p-value^a^
Length of hospital stay (days)^a^	0.0 (0, 0)	2 (0, 6)	0.001
Time until CT was ordered from admission (hours)^a^	0 (0, 0)	16 (9, 18)	0.001
Surgery consult placed	47 (98%)	67 (83%)	0.009
CT ordered before surgical consult (%)	1 (2%)	43 (65%)	0.001
CT ordered after surgical consult (%)	1 (2%)	14 (17%)	0.009
Patient follow-up in surgery clinic (%)	19 (43%)	55 (68%)	0.007
Patient discharged from ED (%)	23 (85%)	34 (42%)	0.001

## Discussion

Perianal abscesses and other infectious disorders of the anorectum are commonly present in a wide variety of clinical settings, including emergency departments, primary care physicians' offices, and urgent care. Although perianal abscesses are common, their exact incidence is unknown [5]. The patient population that may present with perianal abscesses is diverse and includes patients with chronic symptoms or other underlying disease processes [7]. Patients with perianal abscesses may present with throbbing pain, visible swelling, and redness of the anus. Physical examination often reveals an area that is tender to palpation and possibly fluctuant [8]. When patients present to the emergency department with a suspected perianal abscess, a variety of imaging modalities may be used for initial evaluation [9].

Imaging may be used when a perianal abscess is suspected to eliminate other differential diagnoses, guide surgical management, predict surgical outcomes, assess residual disease, and monitor the efficacy of medical therapy [4]. CT is commonly used in acute settings to evaluate perianal abscesses; however, the inherent lack of contrast resolution on CT limits the ability to differentiate between small abscesses and fistulas from the anal sphincter and soft tissue of the pelvic floor [10]. The reported sensitivity of CT for perianal abscesses is 77% [5]. When comparing CT and endoanal ultrasound, CT was able to correctly classify 24% of perianal abscesses, while endoanal ultrasound correctly classified 82% [5].

From the data collected in this retrospective review of patients at a single county institution, no significant differences (p>0.05) were found in the ultimate diagnosis of perianal abscess between patients who underwent CT imaging performed and those who did not have CT imaging performed across different variables such as age, sex, race, and concomitant risk factors such as diabetes mellitus, hypertension, hyperlipidemia, chronic kidney disease, and congestive heart failure. This demonstrates the appropriate stratification across the groups. Significant differences were found between patients who underwent CT imaging and those who did not have CT imaging performed in various aspects of clinical management, including length of stay (LOS) inpatient, time until CT was ordered from admission, surgery consults being placed, follow-up in surgery outpatient clinic, and discharge from the emergency department.

Hospital LOS was defined as the number of days a patient resided in the hospital, from the day of admission to the day of discharge. LOS is a key performance indicator used widely across developed countries to assess hospital efficiency to be better able to optimize healthcare costs without compromising the quality of patient outcomes [11]. LOS is not only associated with increased costs but also with an increased rate of medical conditions that further delay discharge. A longer LOS decreases the number of available acute beds and creates bottlenecking within the hospital, decreasing turnover rates. LOS improvements can improve hospital system capacities, such as beds and staff time, which would allow for better allocation of hospital resources [3].

Increases in LOS can be attributed to various factors, including delayed diagnostic services and the results of investigations. Additional contributing factors may include limited knowledge of best practices or a fear of liability. These behavioral, clinical, and operational factors give rise to the notion of "waste," as identified by the PricewaterhouseCoopers 2008 report, which accounts for more than half of the total healthcare spending in the United States. Achieving an optimized balance between the efficient use of hospital resources and centering on patient needs is a significant challenge in health care. Therefore, understanding the factors contributing to an increased LOS is of great importance [3].

The results of this study found no significant difference between having a CT performed or not having a CT performed on a patient's ultimate diagnosis of perianal abscess, but found significant differences in hospital outcomes such as LOS. Patients who underwent CT had longer LOS. The findings of this study suggest that patients presenting with a suspected perianal abscess may still be successfully diagnosed and managed appropriately with a shorter length of stay using clinical history and physical examination. Recommended practices suggest a clinical exam is the appropriate first line on diagnosis. 

The limitations of this retrospective study include those inherent to a retrospective study, unrecorded outcomes by clinicians, unavailability of data on confounders that may lead to bias, a single-center review, and unknown confounders. Further research may involve prospective study designs to explore the usefulness and efficacy of CT imaging in cases of suspected perianal abscesses.

## Conclusions

No significant differences were found in the ultimate diagnosis of perianal abscess between patients who did and did not undergo CT imaging across multiple variables. Significant differences were found between patients who did and did not undergo CT imaging across various aspects of clinical management, including wait time in the emergency department, and inpatient LOS. The findings of this study suggest that patients presenting with suspected perianal abscess may still be successfully diagnosed and managed clinically while decreasing emergency department and inpatient LOS if CT imaging is avoided.
